# Cognitive Function Related to the *Sirh11/Zcchc16* Gene Acquired from an LTR Retrotransposon in Eutherians

**DOI:** 10.1371/journal.pgen.1005521

**Published:** 2015-09-24

**Authors:** Masahito Irie, Masanobu Yoshikawa, Ryuichi Ono, Hirotaka Iwafune, Tamio Furuse, Ikuko Yamada, Shigeharu Wakana, Yui Yamashita, Takaya Abe, Fumitoshi Ishino, Tomoko Kaneko-Ishino

**Affiliations:** 1 School of Health Sciences, Tokai University, Isehara, Kanagawa, Japan; 2 Department of Epigenetics, Medical Research Institute, Tokyo Medical and Dental University (TMDU), Bunkyo-ku, Tokyo, Japan; 3 Department of Clinical Pharmacology, Tokai University School of Medicine, Isehara, Kanagawa, Japan; 4 Technology and Development Team for Mouse Phenotype Analysis, The Japan Mouse Clinic, RIKEN BioResource Center (BRC), Tsukuba, Ibaraki, Japan; 5 Animal Resource Development Unit, Division of Bio-function Dynamics Imaging, RIKEN Center for Life Science Technologies (CLST), Chuou-ku, Kobe, Japan; 6 Genetic Engineering Team, Division of Bio-Function Dynamics Imaging, RIKEN Center for Life Science Technologies (CLST), Chuou-ku, Kobe, Japan; 7 Global Center of Excellence Program for International Research Center for Molecular Science in Tooth and Bone Diseases, Tokyo Medical and Dental University, Bunkyo-ku, Tokyo, Japan; University of Utah School of Medicine, UNITED STATES

## Abstract

Gene targeting of mouse *S* *ushi-* *i* *chi-related* *r* *etrotransposon* *h* *omologue* *11* */* *Z* *inc finger* *CCHC* *domain-containing* *16* (*Sirh11/Zcchc16*) causes abnormal behaviors related to cognition, including attention, impulsivity and working memory. *Sirh11/Zcchc16* encodes a CCHC type of zinc-finger protein that exhibits high homology to an LTR retrotransposon Gag protein. Upon microdialysis analysis of the prefrontal cortex region, the recovery rate of noradrenaline (NA) was reduced compared with dopamine (DA) after perfusion of high potassium-containing artificial cerebrospinal fluid in knockout (KO) mice. These data indicate that *Sirh11/Zcchc16* is involved in cognitive function in the brain, possibly via the noradrenergic system, in the contemporary mouse developmental systems. Interestingly, it is highly conserved in three out of the four major groups of the eutherians, euarchontoglires, laurasiatheria and afrotheria, but is heavily mutated in xenarthran species such as the sloth and armadillo, suggesting that it has contributed to brain evolution in the three major eutherian lineages, including humans and mice. *Sirh11/Zcchc16* is the first *SIRH* gene to be involved in brain function, instead of just the placenta, as seen in the case of *Peg10*, *Peg11/Rtl1* and *Sirh7/Ldoc1*.

## Author Summary

Retrotransposon-derived DNA sequences occupy approximately 40% of the mammalian genome, compared with only 1.5% of protein coding genes. They have been commonly considered “junk DNA” and even potentially harmful for host organisms. However, a series of knockout (KO) mouse analyses demonstrated that at least some of the LTR retrotransposon- and retrovirus-derived sequences play essential roles in the current mammalian developmental system as endogenous genes, such as *Peg10*, *Peg11/Rtl1*, *Sirh7/Ldoc1*, *SYNCYTINs* and *FEMATRIN-1*, which are active in multiple aspects of placental function. Here we demonstrate that another LTR retrotransposon-derived gene, *Sirh11/Zcchc16*, plays an important role in cognitive function in the brain. *Sirh11/Zcchc16* KO mice exhibit abnormal behaviors related to cognition, including attention, impulsivity and working memory, possibly due to the locus coeruleus-noradrenaline (LC-NA) system, suggesting that human *SIRH11/ZCCHC16* may be involved in X-linked intellectual disability and/or attention-deficit/hyperactivity disorder. Comparative genome analysis demonstrates that *SIRH11/ZCCHC16* was acquired in a common eutherian ancestor, suggesting that it contributed to eutherian brain evolution because it confers a critically important advantage in the competition that occurs in daily life. This study provides further insight into the impact of LTR retrotransposon-derived genes on mammalian evolution.

## Introduction

Mammals, including human beings, have evolved a unique viviparous reproductive system using a placenta and a highly developed central nervous system. How did these unique characteristics emerge in mammalian evolution? Retrotransposons occupy approximately 40% of the mammalian genome. They recently have attracted attention as one of the driving forces of genomic evolution, providing novel endogenous genes [1–11] as well as rewiring the genetic network in the form of novel *cis*-elements, such as promoters, enhancers, insulators and transcription factor binding sites [12–16].

In a series of KO mouse experiments we have demonstrated that at least three LTR retrotransposon-derived genes are essential for mammalian development and reproduction via multiple placental functions; *Peg10* is involved in the formation [8] and *Peg11/Rtl1* in the maintenance of the placenta [9], while *Sirh7/Ldoc1* is involved in endocrine regulation via the differentiation/maturation of a variety of placental cells [11], suggesting that they all have profoundly contributed to the evolution of viviparity during mammalian evolution [8–11, 17, 18].

Two major families of genes derived from Ty3/Gypsy LTR retrotransposons have been identified: one is the *SIRH* family, comprising the 11 genes mentioned above, while the other is the paraneoplastic MA antigen (*PNMA*) family derived from a gypsy_12DR-related retrotransposon comprised of at least 19 and 15 genes in humans and mice, respectively [7, 19–22]. It should be noted that *Peg10* is the only gene commonly conserved in both the eutherians and marsupials [23], while all the others exist as eutherian- or marsupial-specific genes [11, 22, 24, 25]. Among the *SIRH* family, *Sirh11/Zcchc16* (also called *Mart4*) is unique because it does not exhibit any placental expression during development, but rather, is expressed in the brain, testis, ovary and kidney.

In this work, we set out to examine whether *Sirh11/Zcchc16* plays a role in organs other than the placenta, then generated and analyzed *Sirh11/Zcchc16* KO mice. Interestingly, the *Sirh11/Zcchc16* KO mice exhibited a variety of behavioral abnormalities related to cognition, indicating *Sirh11/Zcchc16* is involved in brain function. We also found abnormal regulation of the NA level in the prefrontal cortex of KO mice. As the noradrenergic system in the LC in the brainstem sends projections to virtually all brain structures, including the prefrontal cortex of the cerebrum, and has been proposed to be involved in cognitive function, such as impulsivity, attention, working memory and their associated behaviors in mammals [26–29], we investigated the potential role of the Sirh11/Zcchc16 protein in the noradrenergic system, suggesting the relationship to human mental disorders and the impact on brain evolution in eutherian mammals.

## Results

### *Sirh11/Zcchc16* was acquired from a common ancestor of the eutherian mammals

**Fig 1:**
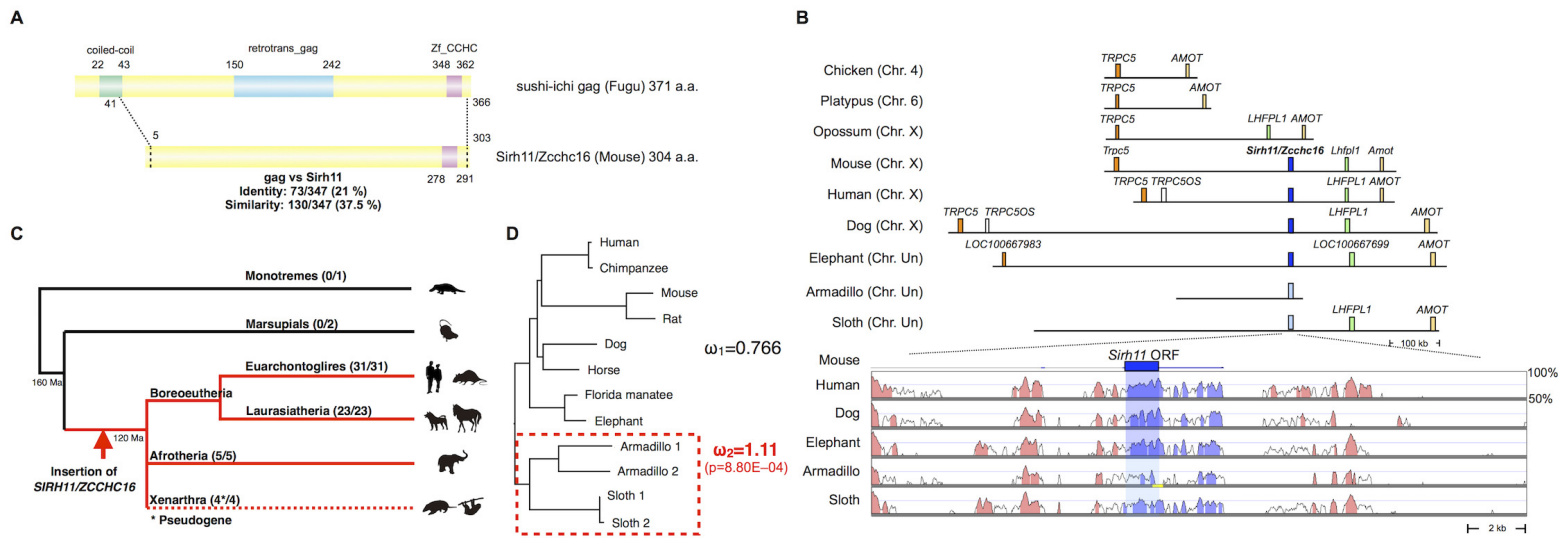
Characteristics of Sirh11/Zcchc16. (A) Structure of the Sirh11/Zcchc16 protein. The scheme shows the alignment of the sushi-ichi gag domain and Sirh11/Zcchc16 proteins, which overall exhibit 21% identity and 37.5% similarity, respectively. The zinc finger CCHC domain (purple) in the C-terminus is conserved, but the coiled-coil motif (green) and gag domain (blue) are absent in the Sirh11/Zcchc16 protein. (B) Chromosomal Location. *SIRH11/ZCCH16* is conserved in an orthologous chromosomal region between *TRPC5* and *LHFPL1* in eutherian mammals. The upper panel shows that *SIRH11/ZCCHC16* is absent in the chicken (birds), platypus (monotremes) and opossum (marsupials). The identically colored boxes represent orthologous genes and the light blue boxes represent *psuedoSIRH11/ZCCHC16* genes. The lower panel provides a comparison of *SIRH11/ZCCHC16* and its flanking genome sequences with 50~100% homology to the mouse genome in several eutherian mammals. The purple (*SIRH11/ZCCHC16*) and red (others) areas indicate evolutionarily conserved sequences (ECSs). The shaded area indicates the regions that correspond to the mouse *Sirh11/Zcchc16* open reading frame. The yellow line in the Armadillo column represents the gap region in the genome sequence. (C) Conservation of *Sirh11/Zcchc16* in eutherian mammals. The *SIRH11/ZCCHC16* sequence was confirmed in four major eutherian groups (red), euarchontoglires, laurasiatheria, xenarthra and afrotheria, but became a pseudogene in xenarthra (the dashed line), indicating that the insertion of *SIRH11/ZCCHC16* occurred in a common eutherian ancestor. The number of species possessing *SIRH11/ZCCHC16* (front) and those which in total were analyzed (back) are noted in parentheses. (D) PAML analysis. Two models, the one-ratio model based on the assumption that ω_1_ (dN/dS) is the same for all of the branches (model 1) and the two-ratio model based on the assumption that ω_2_ in the xenathran lineage is different from ω_1_ in all the others (model 2) were compared. The model 2 was statistically significant and is shown. The *p*-value was calculated by the likelihood ratio test. Armadillo 1 and 2 represent *Dasypus novemcinctus* and *Tolypeutes matacus*, respectively. Sloth 1 and 2 represent *Choloepus hoffmanni* and *Choloepus didactylus*, respectively.

Mouse *Sirh11/Zcchc16* encodes a Gag-like protein comprising 304 amino acids with a typical CCHC RNA-binding motif at the C-terminus (Fig 1A). It exhibits 37.5% homology with the entire sushi-ichi retrotransposon Gag, consisting of 371 amino acids, except for the N-terminus. *Sirh11/Zcchc16* is located on the X chromosome between *Trpc5* and *Lhfpl1*. Its location is conserved in all of the eutherian lineages, euarchontoglires, laurasiatheria, afrotheria and xenarthra (Fig 1B and 1C). However, it became a pseudogene by frameshift and nonsense mutation in xenarthran species, such as the armadillo and sloth (S1 Fig). In the case of the armadillo (*Dasypus novemcinctus*), contig including *pseudoSIRH11/ZCCHC16* is short and thus does not reach *LHFPL1* or *TRPC5*. However, the presence of several evolutionarily conserved sequences (ECSs) in its surrounding 20 kb sequence confirms that it is orthologous to *SIRH11/ZCCHC16* (Fig 1B, lower column). The absence of *SIRH11/ZCCHC16* from marsupials, monotremes and birds was also confirmed, because there is no orthologous gene between *TRPC5* and *LHFPL1* in the opossum and Tasmanian devil, or between *TRPC5* and *AMOT* in the chicken and platypus, respectively (Fig 1B and 1C). This indicates that the insertion of *SIRH11/ZCCHC16* occurred in a common eutherian ancestor after the spilt of the eutherians and marsupials 160 million years ago (Ma), before the diversification of the three major eutherian lineages, boreoeutheria (including euarchontoglires and laurasiatheria), afrotheria and xenarthra, 120 Ma [30].

Importantly, the dN/dS ratio in the pairwise comparisons of *SIRH11/ZCCHC16* orthologs between mouse and seven eutherian species other than xenarthran species is approximately 0.35~0.45 (< 1) (Table 1)[31], suggesting that *SIRH11/ZCCHC16* has been subjected to purifying selection after its domestication (exaptation) in the common eutherian ancestor. Furthermore, to examine whether the functional constraint is relaxed in the xenarthran lineage, we also performed an analysis using the Phylogenetic Analysis by Maximum Likelihood (PAML) program for comparing two models [31]. One is that all the twelve species including the four xenarthran species have the same ω value (dN/dS ratio) (model 1). The other is based on the assumption that the ω value in the xenarthran branch, including the two armadillo and two sloth species, is different from that of all the other eight eutherian species (model 2). The analysis demonstrated that model 2 is statistically significant (p = 8.80E-04): ω_2_ = 1.11 for the xenarthran branch, with ω_1_ = 0.766 for all of the others (Fig 1D), that is, relaxed or neutral evolution is ongoing in xenarthra. All of these results indicate that *SIRH11/ZCCHC16* is a protein-coding gene in the eutherians except in xenarthra.

**Table 1:** The dN/dS ratio between the mouse and the seven other eutherian species expect xenarthral.

dN/dS rario	Rat	Human	Chimpanzee	Dog	Horse	Manatee	Elephant
Mouse	0.3858	0.3947	0.4026	0.3541	0.4542	0.4339	0.392

Pairwise dN/dS analysis was performed using PAML [31].

### *Sirh11/Zcchc16* KO mice exhibited normal development and postnatal growth

**Fig 2:**
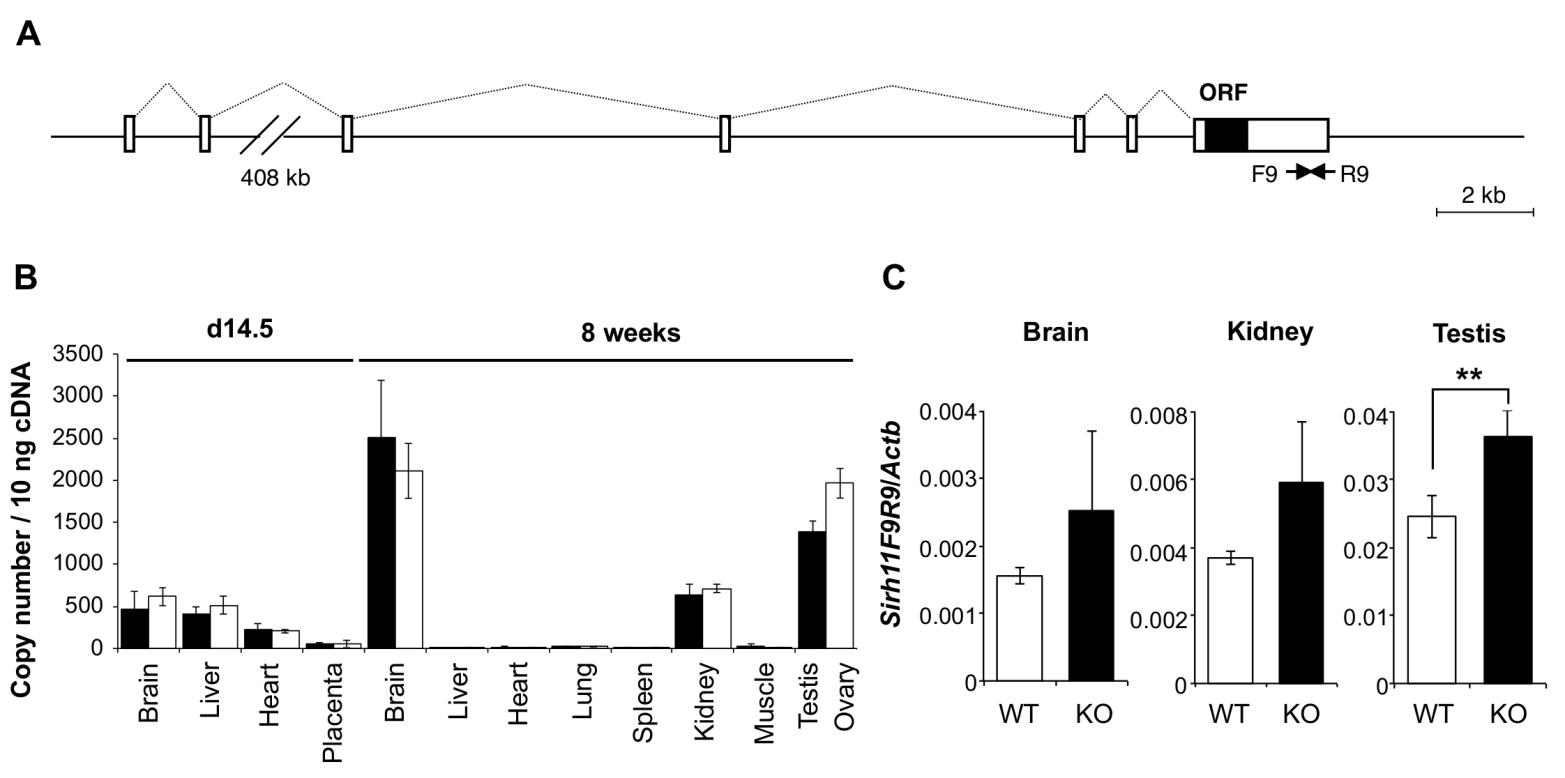
Expression profile of mouse *Sirh11/Zcchc16* mRNA. (A) Exon-intron structure of the *Sirh11/Zcchc16* gene. The full-length cDNA sequence was identified by 5'- and 3'-RACE (Rapid Amplification of cDNA Ends) experiments using brain RNA at 8 w. The *Sirh11/Zcchc16* gene consists of 7 exons and the protein coding sequence is in exon 7. The identified cDNA sequence corresponds to Genbank Accession No. NM_001033795.4. The white and black boxes represent the exons and ORF, respectively. (B) *Sirh11* expression in fetuses and adults. qRT-PCR analyses were carried out using C57BL/6J cDNA from various tissues and organs at d14.5 and 8 w. The black and white bars indicate males and females, respectively. Data represents the mean ± S. D. (N = 3 each). *Sirh11/Zcchc16* expression was observed in the brain, testis, ovary and kidney, but not in the placenta. qRT-PCR primers (F9R9) were designed in the 3’ UTR region of *Sirh11/Zcchc16* mRNA (see arrows in Fig 2A). (C) Overexpression of the *Sirh11/Zcchc16* transcript without any ORF in KO mice. The graph shows the *Sirh11/Zcchc16* expression levels at 8 weeks of age using F9R9 (3’ UTR) primer sets relative to *Actb* mRNA in the male brain, kidney and testis. The white and black bars represent WT and KO, respectively. Each data point represents the mean ± S. D. (N = 4 each). The asterisks indicate significant differences between the WT and KO mice (**: p < 0.01).

*Sirh11/Zcchc16* is basically comprised of 7 exons, with its ORF in the last exon (Fig 2A). According to the NCBI database, there are at least three variants with different first exon sequences, presumably dependent on the tissues and organs where it resides. A low level of *Sirh11/Zcchc16* expression was observed in the brain, liver and heart on embryonic day 14.5 (d14.5), with a moderate level of expression in the brain, kidney, testis and ovary in adults (8 weeks (8 w)) (Fig 2B). We generated *Sirh11/Zcchc16* KO mice using TT2 ES cells by means of a complete deletion of its protein coding sequence (S2 Fig). After removing the neomycin cassette, *Sirh11/Zcchc16* KO mice were backcrossed to B6 more than 10 generations. We confirmed the lack of the ORF region by RT-PCR using a primer set (F5R5). However, it should be noted that the RT-PCR experiment using primer sets amplifying its 3’-UTR region (such as F9R9) exhibited an approximately 1.5 fold increment in these organs in the KO mice (although not significant in the whole brain or kidney), suggesting the existence of a feedback mechanism regulating *Sirh11/Zcchc16* at the protein level (Fig 2A and 2C).

*Sirh11/Zcchc16* KO mice did not exhibit lethality or growth retardation in the pre- and postnatal periods in either female (homo) or male (null) KO mice (Tables 2 and 3). Despite the relatively higher expression of *Sirh11/Zcchc16* in the testis and oocyte, both the male and female KO mice were fertile, even in the case of mating between female homo KOs and null male mice, indicating that *Sirh11/Zcchc16* has no apparent role in sperm and/or egg production (Table 2). No abnormalities were detected in the urine of the *Sirh11/Zcchc16* male KO mice (10 w), such as pH or the amount of glucose, total protein, urobilinogen, ketone body, bilirubin and occult blood, suggesting that kidney function is also normal in these KO mice (S3 Fig).

**Table 2:** Mating experiment.

Mating pair	+/Y	−/Y	+/+	+/−	−/−	Total	Average litter size
♀ B6 × ♂ −/Y	25	-	-	30	-	55	6.9
♀ +/+ × ♂ B6	23	-	19	-	-	42	5.3
♀−/− × ♂ B6	-	30	-	26	-	56	5.1
♀ −/− × ♂ −/Y	-	11	-	-	11	22	5.5

Both the null *Sirh11/Zcchc16* KO (–/Y) and *Sirh11/Zcchc16* homo KO (–/–) mice exhibited normal reproductive ability. The number of pups in each genotype is shown.

**Table 3:** Postnatal growth.

	Age	8w	10w	11w	12w	13w	14w	15w
mean	WT	23.59	25.02	25.55	25.76	26.09	26.57	27.11
	KO	23.98	25.59	26.09	26.41	26.77	27.38	27.85
S.D.	WT	1.33	1.29	1.28	1.26	1.46	1.42	1.67
	KO	1.74	1.80	2.13	2.30	2.50	2.61	2.60
	p	0.321	0.155	0.230	0.184	0.226	0.160	0.243
N	WT	22	22	21	19	18	18	16
	KO	40	40	38	35	33	33	32

*Sirh11/Zcchc16* KO mice exhibited normal growth. The mean body weight (grams) in the male mice is shown.

### Behavioral abnormalities observed in *Sirh11/Zcchc16* KO mice

**Fig 3:**
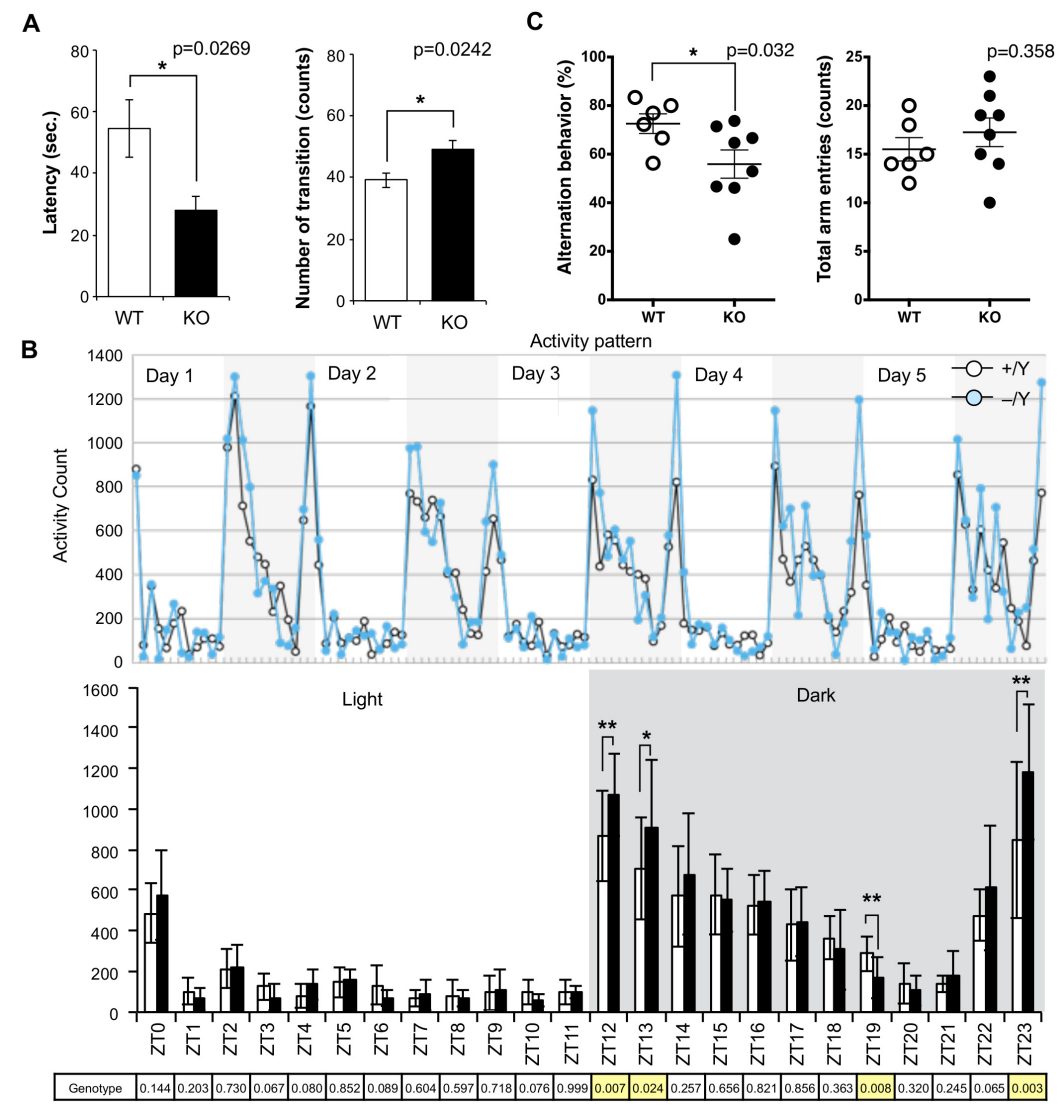
Abnormal behavior in the *Sirh11/Zcchc16* KO mice. **(**A) Light/Dark transition test. The left panel shows the latency time before entering into the light chamber. The right panel shows the number of transitions. The white and black bars represent WT and KO, respectively. Each data point represents the mean ± S. E. M. (N = 7 each). The asterisks indicate significant differences between the male WT and KO mice (*: p < 0.05). **(**B) Home-cage activity test. Upper: The plots show the activity counts every hour over 5 days. The white and grey areas indicate the light and dark phases, respectively. Middle: The white and black bars represent the activity counts in the WT and KO, respectively (mean ± S. D. (N = 7 each)). Zeitgeiber time (ZT) is shown on the x-axis. The asterisks indicate significant differences between the male WT and KO mice (**: p < 0.01, *: p < 0.05). Lower: the table shows the p-values of the two-way ANOVA at each ZT. The yellow columns indicate a significant difference in genotype (p < 0.05). (C) Y-maze test. Left: each plot shows the percentage of alternation behavior. Right: each plot shows the number of total arm entries. The white and black plots represent the WT and KO, respectively. The asterisk indicates a significant difference between the male WT (N = 6) and KO (N = 8) mice (*: p < 0.05).

Although no evident structural abnormalities were found in the KO mouse brain (S4 Fig), it was noticed that abnormal behaviors were exhibited. For example, they displayed agitated movement in their cages when staff personnel entered the breeding room, and sometimes jumped out when their cages were exchanged. Therefore, comprehensive behavior tests were carried out using 8–10 w males. *Sirh11/Zcchc16* KO mice exhibited no abnormality in the open field test (9 and 10 w), but in the Light/Dark transition test (8 w) the latency before entering into the light chamber was significantly decreased (unpaired two sample *t*-test, *t*(12) = 2.52, p = 0.0269), while the number of transitions was significantly increased (unpaired two sample *t*-test, *t*(12) = -2.58, p = 0.0242) compared to the wild type (WT), suggesting a reduced attention and/or enhanced impulsivity (Fig 3A). They also exhibited significantly higher activity during the dark period of the home-cage activity test (10 w), especially just after the “light to dark (Zeitgeiber time (ZT) 12 and 13)” (effect of genotype, *F*(1, 60) = 7.86, p = 0.00681 and effect of genotype, *F*(1, 60) = 5.35, p = 0.0241, respectively) as well as just before the “dark to light (ZT23)” (*F*(1, 60) = 9.77, p = 0.00274) transition periods. There was also lower activity at ZT19 (*F*(1, 60) = 7.56), p = 0.00788), while there was no significant difference in the light periods (ZT0-ZT11), suggesting hyperactivity, especially when the light conditions are changed or about to be changed (Fig 3B).

Next, the Y-maze test was conducted to assess spatial working memory by recording spontaneously alternating search behavior during a 5-min session in a Y-maze (16–18 w). It is considered that alternating search behavior reflects a primitive working memory capacity because it is based on the tendency of normal animals to enter the arm of the Y-maze which was least recently explored [32]. The Y-maze test also allows the simultaneous assessment of hyperactivity independently of spatial working memory. Hyperactivity may interfere with learning and memory, therefore, its assessment is crucial for the interpretation of memory test results [33]. Importantly, KO mice exhibited a lower level of alternation (Mann-Whitney U test, p = 0.032), although the total number of arm entry events was the same (Mann-Whitney U test, p = 0.358) as the WT controls, suggesting that they have a poor working memory (Fig 3C). Some of the KO mice jumped out of the Y-maze stage before the test started, while none of the control mice exhibited such behavior. Although we excluded this data from the results, this also appears to indicate that these KO mice tend to exhibit extreme behavior when transferred to a new environment. From these results, we reasoned that *Sirh11/Zcchc16* KO mice have some abnormality in cognition, possibly related to monoamine function in the brain, that impacts impulsivity, attention and/or memory.

We used null KO male mice for behavioral tests. Although *Sirh11/Zcchc16* is also expressed in the kidney, testis and ovary, there was no evidently unusual phenotype in these organs and also no viability or growth effects observed, as mentioned. Therefore, we think that the abnormal behaviors observed in the KO mice mainly reflect an impairment of brain function. In addition, we used mice generated by *in vitro* fertilization (IVF) between KO males and hetero KO females for these behavioral tests, as described in Materials and Methods. The fertilized eggs were transferred to the pseudopregnant ICR females, after which the pups were born naturally and taken care of by the ICR mothers. Thus, harmful effects on the pups from the potentially abnormal KO mothers in terms of their nursing behavior were completely excluded. Therefore, we believe that these results reflect a difference in genotype.

### Change of the NA level in the prefrontal cortex of *Sirh11/Zcchc16* KO mice

**Fig 4:**
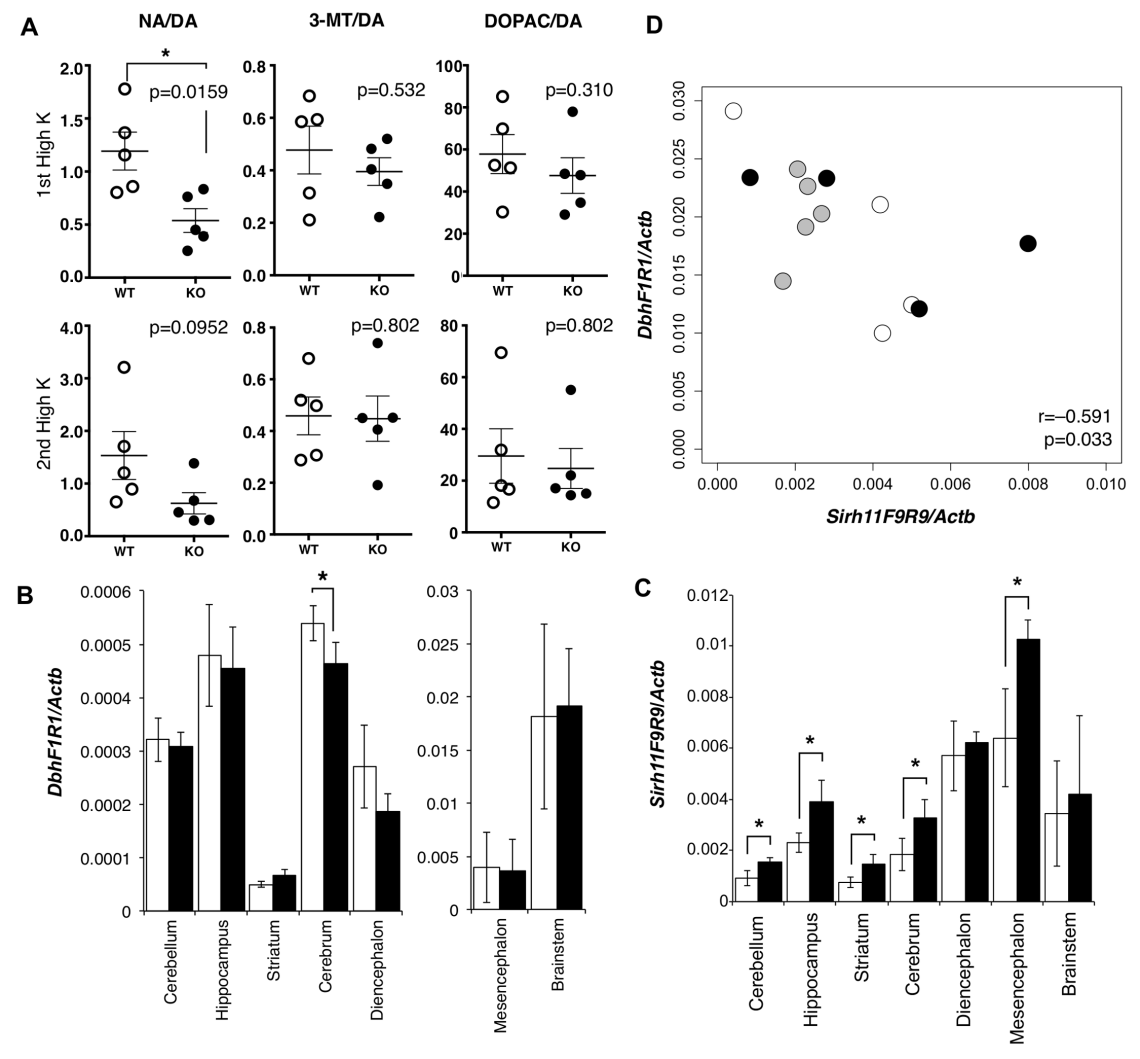
Abnormality in brain of *Sirh11/Zcchc16* KO mice. (A) Microdialysis analysis in the prefrontal cortex in the cerebrum. The levels of various monoamines, including DA, NA, 3-MT and DOPAC were measured after perfusion of high potassium-containing artificial cerebrospinal fluid in the prefrontal cortex. Each plot shows the ratio of the DA metabolites, NA, 3-MT and DOPAC, to DA. The asterisk indicates a significant difference between the WT and KO mice (*: p < 0.05). The three lines on the plots indicate the mean ± S. E. M. (B) *Dbh* mRNA expression in each part of the brain. The white and black bars represent the relative expression levels of *Dbh* to *Actb* mRNA in WT and KO, respectively (mean ± S. D., N = 4 each). The asterisk indicates a significant difference between the WT and KO (*: p < 0.05). The expression levels in the mesencephalon and brainstem are shown in a separate figure with a different scale. **(**C) *Sirh11* mRNA expression in each part of the brain. The white and black bars represent the relative expression levels of *Sirh11* (3’ UTR) to *Actb* mRNA in the WT and KO mice, respectively (mean ± S. D., N = 4 each). The asterisk indicates a significant difference between the WT and KO mice (*: p < 0.05). (D) Negative correlation between *Sirh11/Zcchc16* and *Dbh* mRNA expression levels in the brainstem. The plots show the relative expression levels of *Sirh11* (x-axis) and *Dbh* (y-axis) to *Actb* mRNA in the brainstem. The white, black and grey circles indicate the WT, KO and B6, respectively. The Pearson correlation coefficient (r) is shown in the plots. The p-value was calculated by the test for non-correlation.

We carried out microdialysis analysis in the prefrontal cortex of the cerebrum to directly examine the monoamine levels in the KO brain [34–35] because it is well documented that prefrontal cortical NA as well as dopamine (DA) plays an important role in spatial working memory [36]. The levels of various monoamines were measured, mainly at 11~13 w of age, including DA, NA, adrenaline (AD), 3-methoxytyramine (3-MT), 5-hydroxyindole acetic acid (5-HIAA), serotonin (5-HT), 3, 4-dihydroxyphenylacetic acid (DOPAC), homovanillic acid (HVA), 3-methyl-4-hydroxy-phenylglycol (MHPG) and normetanephrine (NM) using isoproterenol (ISO) as the control. During overnight experiments for long-term recording, we found that these levels varied suddenly and unexpectedly, presumably because of abrupt changes in sound and vibration in the experiment room. The levels were also greatly dependent on the initial condition of the mice. As it was difficult to obtain reproducible data in the normal experimental setting, we adopted the perfusion method to overcome this difficulty. Perfusion by high-K solution intensively stimulates the release of neurotransmitters and hence enables an evaluation of the sum of neurotransmitter content at the synapse and in the neuron. High K solution was applied twice, with a 120-min interval. The release of neurotransmitter in response to the second perfusion of the high K solution is dependent upon the catabolic activity during a 120 min period. It was demonstrated that the recovery of the NA level was significantly delayed compared with the DA level (Mann-Whitney U test, p = 0.0159), while the levels of two other DA metabolites, DOPAC and 3-MT (Mann-Whitney U test, p = 0.532 and 0.310, respectively), were unchanged in the KO mice (Fig 4A).

DA is catabolized in three different pathways to NA, DOPAC and 3-MT by the enzymes dopamine beta monooxigenase (Dbh), monoamine oxidase (MAO) and catechol-O-methyltransferase (COMT), respectively [37]. *Dbh* exhibits a dominant expression pattern in the brainstem, approximately 32 fold greater than the cerebrum (Fig 4B) although its brainstem level was not affected in the *Sirh11/Zcchc16* KO. *Sirh11/Zcchc16* also exhibited a relatively higher expression in the mesencephalon, diencephalon and brainstem compared with other parts of the brain (Fig 4C). Interestingly, both the *Dbh* and *Sirh11/Zcchc16* expression levels fluctuated in the brainstem, but their expression patterns exhibited a negative correlation (i.e. the test for non-correlation, Pearson correlation coefficient (r) = −0.591, p = 0.033, Fig 4D). Therefore, it is possible that they are reciprocally regulated by the same environmental signals. Although the precise biological role of *Sirh11/Zcchc16* as well as the relationship to *Dbh* in the brain are presently unknown, all of these data suggest that behavioral defects of the *Sirh11/Zcchc16* KO mice are somehow related to a dysregulation of the noradrenergic system in the brain.

We used two cohorts of mice, one for the comprehensive behavioral tests, including biochemical analyses, and the other for expression analysis in a range from embryos to adult tissues, as well as microdialysis and the Y-maze test. We believe that the results of the monoamine analysis (microdialysis) accurately reflect the Y-maze test. They also are consistent with the Light/Dark transition and home-cage activity tests in the first cohort of mice.

## Discussion

It has been proposed that the LC-NA system plays an important role in cognitive function, presumably by regulating the balance between focused versus flexible responding, or selective versus scanning attention [25–28]. In the phasic mode, LC cells exhibit selective phasic activation for target stimuli, but only a moderate level of tonic discharge, leading to excellent performance on the specific task with few errors and focused, selective attention. In the tonic mode, the LC cells fail to respond phasically to any task stimuli, but rather, exhibit higher levels of ongoing tonic activity, leading to poor performance with many errors and a form of scanning, labile attention. In their overarching theory, Bouret and Sara proposed that phasic activation of the NA neurons of the LC takes place in time with the cognitive shifts that facilitate dynamic reorganization of target neural networks, permitting rapid behavioral adaptation to the demands of changing environmental imperatives [27].

In this work, *Sirh11/Zcchc16* KO mice exhibited certain clearly evident behavioral abnormalities, such as increased activity during the light/dark transition test, higher daily activities in the period just after dark as well as just before light and a lower score in alternating search behavior in the Y-maze test, indicating that *Sirh11/Zcchc16* plays a role in cognition. Together with certain other sudden and unexpected movement activity that was observed, it is likely that *Sirh11/Zcchc16* KO mice tend to exhibit behaviors related to the classic tonic LC mode. In support of this idea, a significantly lower recovery rate of NA compared to DA in the prefrontal cortex was observed in a microdialysis analysis of the KO mice after perfusion with high potassium solution.

Importantly, activation of the LC-NA system is also associated with an increased accuracy of the response to task-relevant stimuli [26]. Using microdialysis analysis, Rossetti and Carboni demonstrated that both prefrontal cortical DA and NA are involved in the modulation of working memory [36]. From an application of the T-maze test using rats that analyzes such function along with memory and spatial learning via an application of various stimuli, they demonstrated that the prefrontal cortical DA and NA dialysate levels are both phasically increased when rats perform correctly in a delayed alternation task in a T-maze. Together with the findings from other experiments, they ultimately concluded that DA is primarily associated with reward expectancy, whereas NA is involved in the active maintenance of goal-related information as well as the rules for realizing the goal [36]. This is in good accord with the results of our Y-maze test, indicating that *Sirh11/Zcchc16* KO mice with a lower NA recovery rate in the neurons of the prefrontal cortex have impaired spatial working memory.

It has also been proposed that the phasic as opposed to tonic LC activity participates in certain critically important normal behavioral functions as well as severe mental problems, including attention-deficit/hyperactivity disorder (ADHD) and a variety of emotional and affective disorders [25–28]. Therefore, the behavioral abnormalities observed suggest a possible role for *SIRH11/ZCCHC16* in mental disorders.

Although a relatively higher expression level was observed in the brainstem, diencephalon and mesencephalon in the RT-PCR experiment, we have no direct evidence at present as to precisely where *Sirh11/Zcchc16* is expressed in the brain or whether the putative Sirh11/Zcchc16 protein in the NA neurons from the LC. This is despite numerous attempts using *in situ*, Western blotting and immunostaining. However, the conservation of the amino acid sequence of the Sirh11/Zcchc16 in both boreoeutheria and afrotheria clearly demonstrates that it is subjected to purifying selection (dN/dS <1), providing indirect but nonetheless supportive evidence that *Sirh11/Zcchc16* is a protein-coding gene (Table 1). This conclusion is consistent with the finding that the xenarthran *pseudoSirh11/Zcchc16* bearing many mutations in its coding frame has been subjected to relaxed or neutral evolution (dN/dS ~1) (Fig 1D). The Sirh11/Zcchc16 protein possesses a conserved CCHC zinc finger domain with potential RNA-binding capability. Therefore, it is possible that it functions as a part of messenger ribonucleoprotein particles (mRNPs) in neurons because mRNAs synthesized in the soma are transported to neurites and/or synapses as mRNPs by binding to RNA-binding proteins and are translated there [38–40]. However, since the possibility that *Sirh11/Zcchc16* functions as a non-coding RNA has not been completely excluded, these issues need to be further addressed in more detail in the future.

Human *SIRH11/ZCCHC16* is located on Xq23, where several X-linked intellectual disability (XLID) genes have been mapped, such as *PRPS1* (–5 Mb from *SIRH11/ZCCHC16*), *ACSL4* (–2.5 Mb), *PAK3* (–1 Mb), *DCX* (–1 Mb), *AGTR2* (+4 Mb), *LAMP2* (+8 Mb) and *GRIA3* (+11 Mb). As some of the genes responsible for XLID remain to be identified in this chromosomal region, *SIRH11/ZCCHC16* may also be a good candidate for XLID [41, 42]. Interestingly, Cho *et al*. recently reported that some XLID patients have mutations in the *SIZN1/ZCCHC12* (*PNMA10*) gene that locates approximately 6 Mb downstream of *SIRH11/ZCCHC16* and indicated this gene to be a good candidate for XLID [43]. *PNMA10/ZCCHC12* is another eutherian-specific Ty3/Gypsy LTR retrotransposon-derived gene that is known to be involved in transcriptional regulation [44]. Mouse *Pnma10/Zcchc12* is expressed in the embryonic ventral forebrain in a cholinergic-neuron-specific manner and binds to SMAD family proteins. It also acts as a transcriptional co-activator for bone morphogenic protein (BMP) signaling [44]. Both *Sirh11/Zcchc16* and *Pnma10/Zcchc12* encode Gag-like proteins with the CCHC zinc-finger motif, so it will be of interest to determine whether these two retrotransposon-derived proteins have different activities which function in a noradrenergic- and a cholinergic-neuron-specific manner, respectively, in the extant eutherian mammals.

Comparative genome analysis clearly demonstrated that *SIRH11/ZCCHC16* is highly conserved in the three major groups of eutherian mammals, but not in xenarthra, strongly implying that the presence of *Sirh11/Zcchc16* is beneficial in these three eutherian groups, including humans and mice. Our results suggest that *SIRH11/ZCCHC16* contributed to the evolution of the brain by modulating the NA neuronal network in a complex manner. The noradrenergic system is also conserved in other vertebrates, such as fish, amphibians, reptiles and birds, and the general system characteristics are strikingly preserved across a wide range of phylogenetic groups [27]. Therefore, the biological function of the Sirh11/Zcchc16 protein is of great interest in terms of elucidating the evolution of the neuromodulatory system of the brain in the eutherian mammals. What was the function that was replaced in the noradrenergic system by domestication of *SIRH11/ZCCHC16* in these three eutherian lineages, thus permitting rapid behavioral adaptation to changing environmental imperatives? Do the xenarthran species, i.e. the armadillos and sloths, have diminished and/or distinct cognitive activity related to *SIRH11/ZCCHC16*? Although a variety of issues of this type require future investigation, this is the first demonstration that one of the *SIRH* genes plays a role in cognitive function in the brain, presumably via the noradrenergic system.

We previously demonstrated that the three *SIRH* genes, such as *Peg10/Sirh1*, *Peg11/Rtl1/Sirh2* and *Sirh7/Ldoc1*, play essential roles in eutherian development and reproduction, and proposed that these three *SIRH* genes have profoundly contributed to the evolution of viviparity in mammalian evolution as newly acquired genes [8–11]. In addition, the present investigation of *Sirh11/Zcchc16* provides further insight into the impact of LTR retrotransposon-derived genes on the neuromodulatory system in the brain as key step in the evolution of the eutherian mammals.

## Materials and Methods

### Ethics statement

The present animal experiments were performed in strict accordance with the guidelines of Tokai University and Tokyo Medical and Dental University (TMDU), and were approved by the Animal Investigation Committees of Tokai University and TMDU. The open-field test, Light/dark transition test, home-cage activity test and urinalysis were performed in accordance with the guidelines issued by the RIKEN Bioscience Technology Center in their “Outline for Conducting Animal Experiments” (August 1999, revised October 2001).

### Comparative genome analysis

Sushi-ichi gag (AAC33525.1) and mouse Sirh11/Zcchc16 (NP_001028967.2) protein sequences were obtained from NCBI. Amino acid identity and similarity were calculated using the EMBOSS Water program (http://www.ebi.ac.uk/Tools/psa/emboss_water/) in the default mode. The orthologues of SIRH11/ZCCHC16 were identified by search of NCBI Gene (http://www.ncbi.nlm.nih.gov/gene/) using ZCCHC16 as the query term. Genomic homology analysis was performed using the mVISTA LAGAN program (http://genome.lbl.gov/vista/mvista/submit.shtml). We obtained *TRPC5*-*AMOT* genomic sequences from the NCBI database. The sequences used for analysis were the following: Chicken (*Gallus gallus*): gi|358485508:c13102445-12913720; Platypus (*Ornithorhynchus anatinus*): gi|149729612:c11732082-11450308; Opossum (*Monodelphis domestica*): gi|126362945:c69157606-68740637; Mouse (*Mus musculus*): gi|372099090:144381671–145505458; Human (*Homo sapiens*): gi|568815575:111774314–112840908; Dog (*Canis lupus familiaris*): gi|357579592:84841858–85807530; African savanna elephant (*Loxodonta Africana*) gi|343530165:c19381570-18463351; Armadillo (*Dasypus novemcinctus*) gi|476561443; Sloth (*Choloepus hoffmanni*) gi|692243298|gb|KN194663.1|. PseudoSIRH11/ZCCHC16 protein sequences were aligned to Florida manatee SIRH11/ZCCHC16 using Clustal Omega (http://www.ebi.ac.uk/Tools/msa/clustalo/).

### Identification of *pseudoSIRH11/ZCCHC16* in *Tolypeutes matacus* and *Choloepus didactylus*

Genomic DNA was isolated from frozen muscle using the DNeasy Blood & Tissue Kit (QIAGEN). For PCR, the primers were designed at the 5'- and 3'-UTR of pseudoSIRH11/ZCCHC16 using the consensus sequence between *Dasypus novemcinctus* and *Choloepus hoffmanni*. The PCR reaction was performed using Ex*Taq*HS (TaKaRa) with the following conditions: 30 cycles of 98°C, 10 sec; 60°C, 30 sec; 72°C, 1 min. The following PCR primers were used: pseudoSIRH11-F1: 5'-CTTACTGCCTGCCCATTGGT-3' and pseudoSIRH11-R1: 5'-GGATTTTAAAAGTTGGTGCAGG-3'. PCR products were direct-sequenced using the above primers after Exo-SAP-IT (USB) treatment. DNA Data Bank of Japan (DDBJ) accession numbers: LOC064756 for *Tolypeutes matacus SIRH11/ZCCHC16* and LOC064757 for *Choloepus didactylus SIRH11/ZCCHC16*.

### Phylogenic analysis of the *SIRH11/ZCCHC16* gene

A phylogenic tree was constructed with ClustalW2 (Neighbor-joining method) (http://www.ebi.ac.uk/Tools/msa/clustalw2/) using protein coding and pseudo *SIRH11/ZCCHC16* sequences obtained from twelve species. The codon alignment of cDNA was created with the PAL2NAL program (http://www.bork.embl.de/pal2nal/) [45]. The non-synonymous/synonymous substitution rate ratio (ω = dN/dS) was estimated by using CodeML in PAML [31].

### Generation of *Sirh11/Zcchc16* KO mice

To generate *Sirh11/Zcchc16* MT mice (Accession No. CDB0557K: http://www.clst.riken.jp/arg/mutant%20mice%20list.html), we obtained three genomic fragments, the 5’-arm (8 kb: 145111478–145119486; NC_000086), middle arm (1.1 kb: 145119487–145120587; NC_000086) and 3’-arm (2.1 kb: 145120588–145122703; NC_000086) by recombination from the RP23-319K12 BAC clone (BACPAC Resources), and then cloned them into a targeting vector. The targeting vector was introduced into TT2 ES cells (C57BL/6 × CBA genetic background) by electroporation [46]. To generate chimeric mice, ES cells in which homologous recombination had occurred were injected into 8-cell stage embryos. Germ line transmission of the *Sirh11/Zcchc16* MT allele was confirmed by Southern blot and PCR using the genome prepared from pups in which male *Sirh11/Zcchc16* chimeric mice had been crossed with female C57BL/6J. To remove the flox region, we injected a pCAG/NCre plasmid [47] into the fertilized eggs generated by *in vitro* fertilization (IVF) from *Sirh11/Zcchc16* MT hetero eggs and C57BL/6J sperm, thus establishing *Sirh11/Zcchc16* neo mice. To obtain *Sirh11/Zcchc16* KO mice, we injected a pCAGGS-FLPe plasmid (Gene bridge) into the fertilized eggs generated by IVF from *Sirh11/Zcchc16* neo hetero eggs and C57BL/6J sperm. Exclusion of the neo cassette was confirmed by genomic PCR of the pups’ DNA. Southern blot analysis was performed using a standard protocol. Five micro grams of genomic DNA from the tail were digested by restriction enzymes *Nhe*I (for the 5’ probe) and *Nco*I (for the 3’ probe), respectively. Hybond-N+ (GE Healthcare) membranes blotted with digested DNA were hybridized in Church buffer with radio isotope-labelled probes. The 5’ and 3’ probes were generated by genomic PCR using the following sequences: 5’ probe: 145109101–145110193; NC_000086; 3’ probe: 145124154–145124725; NC_000086. The *Sirh11/Zcchc16* KO allele was detected by genomic PCR. Genome DNA was prepared from the tail or ear tip using a DNeasy Blood & Tissue Kit (QIAGEN). PCR was performed using Ex*Taq*HS polymerase (TaKaRa) with the following primers: Sirh11-F1: 5’-ATGTATCCTAAGGTGATCCG-3’ and Sirh11-R2: 5’-ATGTGATGCCACAGCAACTC-3’. *Sirh11/Zcchc16* KO mice were backcrossed to C57BL6/J for more than 10 generations.

### Quantitative RT-PCR

Total RNA was prepared from frozen tissues using ISOGEN (NIPPON GENE) and ISOGEN-LS (NIPPON GENE). The cDNA was made from total RNA (1 μg) using Revertra Ace qPCR RT Master Mix (TOYOBO). Quantitative RT-PCR analysis was performed using Fast SYBR Green Master Mix (Life technologies) and a StepOnePlus System (Life technologies) by means of an absolute quantification method. Data was normalized by *Actb* expression. Student’s *t*-test was used for statistical analysis. The following primer sequences were used: Sirh11-F9: 5’-TGGTGCTGGTGTATTTCCCC-3’ and Sirh11-R9: 5’-TGGCACAGTGGTTAGTGAGGC-3’; Sirh11-F5: 5’-AAGAGGAGGATAGGAAATCACTTTG-3’ and Sirh11-R5: 5’-GTTGTTAGGACAAGGTTGAGG-3’; Dbh-F1: 5’-ACTGAACGGAGAAGCCCTGGAC-3’ and Dbh-R1: 5’-CACCAGAGGACCAACAGGGTCG-3’; Actb-F: 5’-AAGTGTGACGTTGACATCCG-3’ and Actb-R: 5’-GATCCACATCTGCTGGAAGG-3’.

### The mice used for behavioral analyses

To reproduce the hetero and wild-type progeny for the behavior-screen, *in vitro* fertilization (IVF) was performed. Wild type male mice were used as the source for sperm, while hetero female mice were used as the source for the oocytes used for IVF. ICR female mice (CLEA Japan, Tokyo, Japan) were used as pseudopregnant recipients for embryo transfer. Sperm were collected from the *caudae epididymides* of adult male mice (20 w) and allowed to diffuse in human tubal fluid (HTF) medium. After preincubation for approximately 1 hour to allow for capacitation, the sperm were used for insemination. Meanwhile, immature hetero female mice (4 w) were superovulated using intraperitoneal injections of PMSG and HCG (Serotropin and Gonatropin; ASKA Pharmaceutical Co., Tokyo, Japan) with an interval of 48 hours between injections. Approximately 15–17 hours after the HCG injection, the oocyte-cumulus complexes were collected from the oviducts of the superovulated female mice. The complexes from several female mice were then placed in the HTF fertilization medium. Insemination was performed by adding the sperm suspension to the fertilization medium containing complexes and culturing at 37°C with 5% CO_2_ in air. Twenty-four hours after insemination, 2-cell embryos were transferred into the oviducts of pseudopregnant ICR females mated to vasectomized ICR males. All pups were delivered naturally after embryo transfer. After weaning at four weeks age, they were employed as breeding individuals in a single-breeding cage.

### Open-field test

Each mouse was placed in the corner of an open-field apparatus (400 mm wide x 400 mm long x 300 mm high; O’Hara & Co., Ltd., Tokyo, Japan) made of white polyvinyl chloride. The distance traveled by each animal in the open field was recorded for 20 min with a video-imaging system (Image OF9; O’Hara & Co., Ltd., Tokyo, Japan). The mice were tested on two separate occasions at 9 and 10 w. The unpaired two sample *T*-test was used for statistical analysis.

### Light/dark transition test

A commercially available light/dark chamber (O'Hara & Co., Ltd.) was used for the light/dark transition test. The apparatus consists of a light chamber (200 mm long × 200 mm wide × 250 mm high) made of white vinyl chloride plates and a dark chamber with the same dimensions made of black vinyl chloride plates. The apparatus has an opening (50 mm wide × 30 mm high) in the middle of the wall that joins the two chambers. The opening is controlled by a guillotine door. The latency for entering into the lighted chamber and number of transitions between the light and dark chambers were measured. The mice were tested at 8 weeks of age. The unpaired two sample *T*-test was used for statistical analysis.

### Home-cage activity test

Each mouse was placed alone in a testing cage (227 mm wide x 329 mm long x 133 mm high) under a 12-h light–dark cycle (light on at 08:00 h) and had free access to both food and water. After 1 day of acclimation, spontaneous activity in the cage was measured for 5 continuous days (starting at 08:00) with an infrared sensor (activity sensor, O’Hara & Co., Ltd.). The mice were tested at 10 w. The two-way analysis of variance (ANOVA) (effects of genotype and date) was used for statistical analysis.

### Y-maze test

The Y-maze test was performed using male mice at 16–18 weeks of age (WT: N = 6, KO: N = 8). The apparatus was a black, plastic maze with three arms (400 mm long × 30 mm wide × 150 mm high, 120 degrees). Mice were placed at the center of the apparatus and allowed to move freely through the maze for 2 min. The sequence and total number of arm entries were recorded by video camera for 5 min. When all 4 limbs of the mouse were within a pathway, it was considered an entry. An “alternation” was counted when a mouse successively entered 3 different arms. Spontaneous alternating search behavior was calculated by the following equation: alternation behavior (%) = [the number of alternations/(total number of arm entries – 2)] × 100. The results are given as the mean and standard error of the mean (S.E.M.). Statistical analysis was conducted using computer software (Prism, version 6.0c, GraphPad Software, San Diego, CA, USA) for a comparison across the experimental conditions. Statistical evaluations for the measurement of behavior or the NA level were carried out using Mann-Whitney U test. A P-value <0.05 was considered to be significant.

### Microdialysis

We performed *in vivo* microdialysis measurements of extracellular monoamines in the prefrontal cortex of 11–13 week-old mice. A guide cannula (AG-4; EICOM, Kyoto, Japan) was implanted into the prefrontal cortex (+1.9 mm anteroposterior and +0.5 mm mediolateral relative to the bregma and −2.0 mm dorsoventral relative to the dura of the skull) under inhalation anesthesia with nitrous oxide, oxygen and isoflurane (2%). Two days after surgery, a dialysis probe (AI-4-01, 1-mm membrane length; EICOM) was inserted through the guide cannula and perfused at a flow rate of 1 μl/min with artificial cerebrospinal fluid (147.0 mM NaCl, 4.0 mM KCl, 2.3 mM CaCl_2_) or high potassium-containing artificial cerebrospinal fluid (51.0 mM NaCl, 100.0 mM KCl, 2.3 mM CaCl_2_). Samples were collected every 20 min and injected directly into an HPLC column (EICOMPAK CA-5ODS; EICOM) by an auto injector (EAS-20; EICOM). The concentrations of the monoamines in the dialysate were determined by HPLC with an electrochemical detector (ECD300; EICOM). After the experiments, 1 μl of 0.3% Evans blue dye was microinjected through the cannula to histologically verify the position of the probe, and only data from animals with a correct probe placement were used in the analysis. The results are given as the mean and standard error of the mean (S.E.M.) of the data as described in the Y-maze test.

## Supporting Information

S1 Fig Mutations in the pseudoSIRH11/ZCCHC16 protein in the armadillo and sloth. The pseudoSIRH11/ZCCHC16 protein of the two armadillo species (upper) and the two sloth species (lower) were compared with the Florida manatee (afrotheria) SIRH11/ZCCHC16 protein because the Florida manatee *SIRH11/ZCCHC16* exhibits the highest homology to the xenarthran *pseudoSIRH11/ZCCHC16* (61.9% and 79.6% to armadillo and sloth, respectively) at the DNA sequence level. Armadillo 1 and 2 represent *Dasypus novemcinctus* and *Tolypeutes matacus*, respectively. Sloth 1 and 2 represent *Choloepus hoffmanni* and *Choloepus didactylus*, respectively. Note that *Dasypus novemcinctus* (nine-banded armadillo) in the NCBI database lacks information on the C-terminal amino acid sequence. The grey-shaded astrerisks indicate in-frame stop codons. The magenta, blue and orange boxes indicate the sequences translated from +1, +2 and +3 frame of *pseudoSIRH11/ZCCHC16*, respectively. The Xs indicate the frame-shift positions and correspond to certain amino acids depending on the positions. The asterisks, double and single dots below the amino acids indicate identical, highly and slightly similar amino acids to the Florida manatee (afrotheria) SIRH11/ZCCHC16 protein, respectively.
(PDF)

S2 Fig Method of obtaining the *Sirh11/Zcchc16* KO mice. Schematic representation of the WT *Sirh11* locus (WT allele), the targeting vector and targeted *Sirh11/Zcchc16* allele (MT allele), the *Sirh11* ORF allele (black) deleted by Cre recombinase injection (neo allele) and the neo-cassette allele (yellow) deleted by FLPe recombinase injection (KO allele). The red and green triangles represent loxP and frt, respectively. To confirm homologous recombination, Southern blot analysis was carried out using 5’ and 3’ probes (black boxes) in *Sirh11/Zcchc16* MT mice. The genotyping PCR results using the F1 and R2 primers (arrows) are shown. F5, R5, F9 and R9 primers (grey arrows) were used for the qRT-PCR experiment. F5R5 primer sets were designed in the flox region and F9R9 primer sets were in the 3’ UTR.
(PDF)

S3 Fig Urinalysis test performed on the *Sirh11/Zcchc16* KO mice. Bilirubin, glucose, ketone bodies, occult blood, urine protein and urobilinogen as well as the urine pH were examined. Urine-test papers (Aution Sticks 7EA; product code 73507) were purchased from a commercial supplier (Arkray, Tokyo, Japan). The colors of test papers that were immersed to urine were classified by comparing them with standard colors. The horizontal axis of the graph indicates the percentage of mice that exhibited each of the values shown on the right side. The male mice were tested at 10w (N = 7/genotype). There was no significant difference detected between the wild type and knockout mice.
(PDF)

S4 Fig Histological analysis of the brain of the *Sirh11/Zcchc16* KO mice. The brain was isolated from male mice at 8, 12 and 16 w (N = 3 each). The brain was fixed in 4% PFA/PBS overnight and embedded in paraffin. A series of sagittal sections was stained with hematoxylin-eosin (HE). The scale bar represents 1.0 mm. No apparent differences in overall brain structure between the WT and KO were found in any of the stages.
(PDF)
