# Supplementary material for: Cognitive Function Related to the Sirh11/Zcchc16 Gene Acquired from an LTR Retrotransposon in Eutherians
Source: PLoS Genet. 2015 Sep 24;11(9):e1005521. doi: 10.1371/journal.pgen.1005521 (PMC4581854; doi:10.1371/journal.pgen.1005521)
Supplement: S1 Fig — The pseudoSIRH11/ZCCHC16 protein of the two armadillo species (upper) and the two sloth species (lower) were compared with the Florida manatee (afrotheria) SIRH11/ZCCHC16 protein because the Florida manatee SIRH11/ZCCHC16 exhibits the highest homology to the xenarthran pseudoSIRH11/ZCCHC16 (61.9% and 79.6% to armadillo and sloth, respectively) at the DNA sequence level. Armadillo 1 and 2 represent Dasypus novemcinctus and Tolypeutes matacus, respectively. Sloth 1 and 2 represent Choloepus hoffmanni and Choloepus didactylus, respectively. Note that Dasypus novemcinctus (nine-banded armadillo) in the NCBI database lacks information on the C-terminal amino acid sequence. The grey-shaded astrerisks indicate in-frame stop codons. The magenta, blue and orange boxes indicate the sequences translated from +1, +2 and +3 frame of pseudoSIRH11/ZCCHC16, respectively. The Xs indicate the frame-shift positions and correspond to certain amino acids depending on the positions. The asterisks, double and single dots below the amino acids indicate identical, highly and slightly similar amino acids to the Florida manatee (afrotheria) SIRH11/ZCCHC16 protein, respectively. (PDF) [file pgen.1005521.s001.pdf]

```
Armadillo 1        IEKYTESPST-LQLEHFSIWxENLIL*PQVRHQIEENIAPSSQVLPVLATPMMPVPFSLE
Armadillo 2        IKKYAKSPRYLCxLEHFSILAENLILQP*AQHLIEETVAPSSQVLPALATSMMPVPFLLE
FloridaManatee     MEKFTESPPT-LQVEHSSLWADDLILEPQVQHLTDDSTALRGQVMPTLATPMMPVSCSLE
                   ::*:::**     :** *:  ::*** * .:*  ::. *  .**:*.*** ****   **

Armadillo 1        HLTQFHDDHAHFSV-GIPGTTYLTVLKIPSPTDDVQVKLFLITYLRS*RVVGTYLDLTxE
Armadillo 2        HHTQFHDDPANFSV-GSQGTTYLTALKIPNPTDDVQVKFFFDYLSQQMKSCGVLSGPNQx
FloridaManatee     HPSQFHVDPANLSGFLAQATTYLTALKTPNPADDVQVKFFFDYISQQVERCRILPGSD-Q
                   * :*** * *.:*     .*****.** *.*:******:*:    :. .

Armadillo 1        SILVKQYVNFILDFHQSFGKPPK*EMFHLVSTKIDKGDDSSLQYVTFSS---SLLKI*NH
Armadillo 2        STLLKQYENFILEFQESFGKLTKQEMSPVVSSKIDKGDEFSLQYISTFQFHAQNLKLL*K
FloridaManatee     STLLKQYENFVSEFQQSFGEPTKQEMNPLVNAKLDKGNDSFQQYATTFQLLAENISCNKT
                   * *:*** **: :*::***:  * **  :*.:*:***::   ** :  .   . :.

Armadillo 1        YEKSVLRGTGWSNL*QN---EWYTFGQLPDLIIQCIQLNKKHSDRPQLLHSEIQLPQLTF
Armadillo 2        ISSERYRV--VQFVMK*---xRHKFGQPPDLITQCIQLDKKHSDRPELLNAEAQLPRLTF
FloridaManatee     ILSDQLQE-GLADPIQDEVSGTDMMENLPDLITQCIQLDRKRSDRPELLQSEAQLPRLAS
                     ..  :        :         : : **** *****::*:****:**.:* ***:*:

Armadillo 1        LIYHQYFSSPTSTPPKE-------------------------------------------
Armadillo 2        LLYHQYFSSPTSTPPKEETI*LWGGQLPLTTAK*VCQQETQLYSHCSKADQFTRDCFAKH
FloridaManatee     LVHHQYLSSPTGPPAKEEPTQLRGGQLPLTPAKRARQQETQFCLYCSQPGHFTKDCLAKR
                   *::***:****. * **

Armadillo 1        --------------
Armadillo 2        S*ASAKPNN*AH*Q
FloridaManatee     SRAPARSNNPTHQ


Sloth 1            MEKYTESPPSLPVEHF*LQAENLILQPQVQHLNEENTSPSG*VLPALVTPMMPIPFSLEH
Sloth 2            MEKYTESPPSLPVEHF*LQAENLILQPQVQHLNEENTSPSG*VLAALVTPMMPIPFSLEH
FloridaManatee     MEKFTESPPTLQVEHSSLWADDLILEPQVQHLTDDSTALRGQVMPTLATPMMPVSCSLEH
                   ***:*****:* ***  * *::***:******.::.*:  * *: :*.*****:  ****

Sloth 1            LTQFHVDPANFSVG-SQVTTYLTSLKTALNSVDNSQVKSFSDYLSQQMKSCGVLSGPD*S
Sloth 2            LTQFHVDPANFSVG-SQVTTYLTSLKTALNSVNNSQVKSFSDYLSQQMKSCGVLSGPD*S
FloridaManatee     PSQFHVDPANLSGFLAQATTYLTALKT-PNPADDVQVKFFFDYISQQVERCRILPGSDQS
                    :********:*   :*.*****:***  *  .:: *** * **:***:: * :* * * *

Sloth 1            TLLKQYENFIPEYQQSFGKPTKQEMNPVVNAKVDKGNDSSLPLCYHFQFLAQNLSYNETT
Sloth 2            TLLKQYENFIPEYQQSFGKPTKQEMNPVVNAKVDKGNDSSLPLCYHFQFLAQNLSYNETT
FloridaManatee     TLLKQYENFVSEFQQSFGEPTKQEMNPLVNAKLDKGNDSFQQYATTFQLLAENISCNKTI
                   *********: *:*****:********:****:******     .  **:**:*:* *:*

Sloth 1            M*DPLQEGLAGPVCDEMSGKN-MDNPPDLITRCIKLDKKMxSDGPELLQSEVQLPRSTSL
Sloth 2            M*DPLREGLAGPVCDEMSGKN-MDNPPDLITRCIKLDKKMxNDGPELLQSEVQLPRSTSL
FloridaManatee     LSDQLQEGLADPIQDEVSGTDMMENLPDLITQCIQLDRK-RSDRPELLQSEAQLPRLASL
                   : * *:**** *: **:**.: *:* *****:**:**:*  .* *******.**** :**

Sloth 1            IYHQYLSSTTGTLPKEKPV*LQEGQLLLTTAKxAHQQET*FCSYCSQADHFTRDCLTKHS
Sloth 2            IYHQYLSSTTGTLPKEKPV*LQEGQVLLTTGKxAHQQET*FCSYCSQADHFTRDCLTKHS
FloridaManatee     VHHQYLSSPTGPPAKEEPTQLRGGQLPLTPAKRARQQETQFCLYCSQPGHFTKDCLAKRS
                   ::****** **   **:*. *: **: ** .* *:**** ** ****  ***:***:*:*

Sloth 1            RAPAKTNNPACQ
Sloth 2            RAPAKTNNPACQ
FloridaManatee     RAPARSNNPTHQ
                   ****::***: *
```
