# Supplementary figures and images for: Cognitive Function Related to the Sirh11/Zcchc16 Gene Acquired from an LTR Retrotransposon in Eutherians
Source: PLoS Genet. 2015 Sep 24;11(9):e1005521. doi: 10.1371/journal.pgen.1005521 (PMC4581854; doi:10.1371/journal.pgen.1005521)

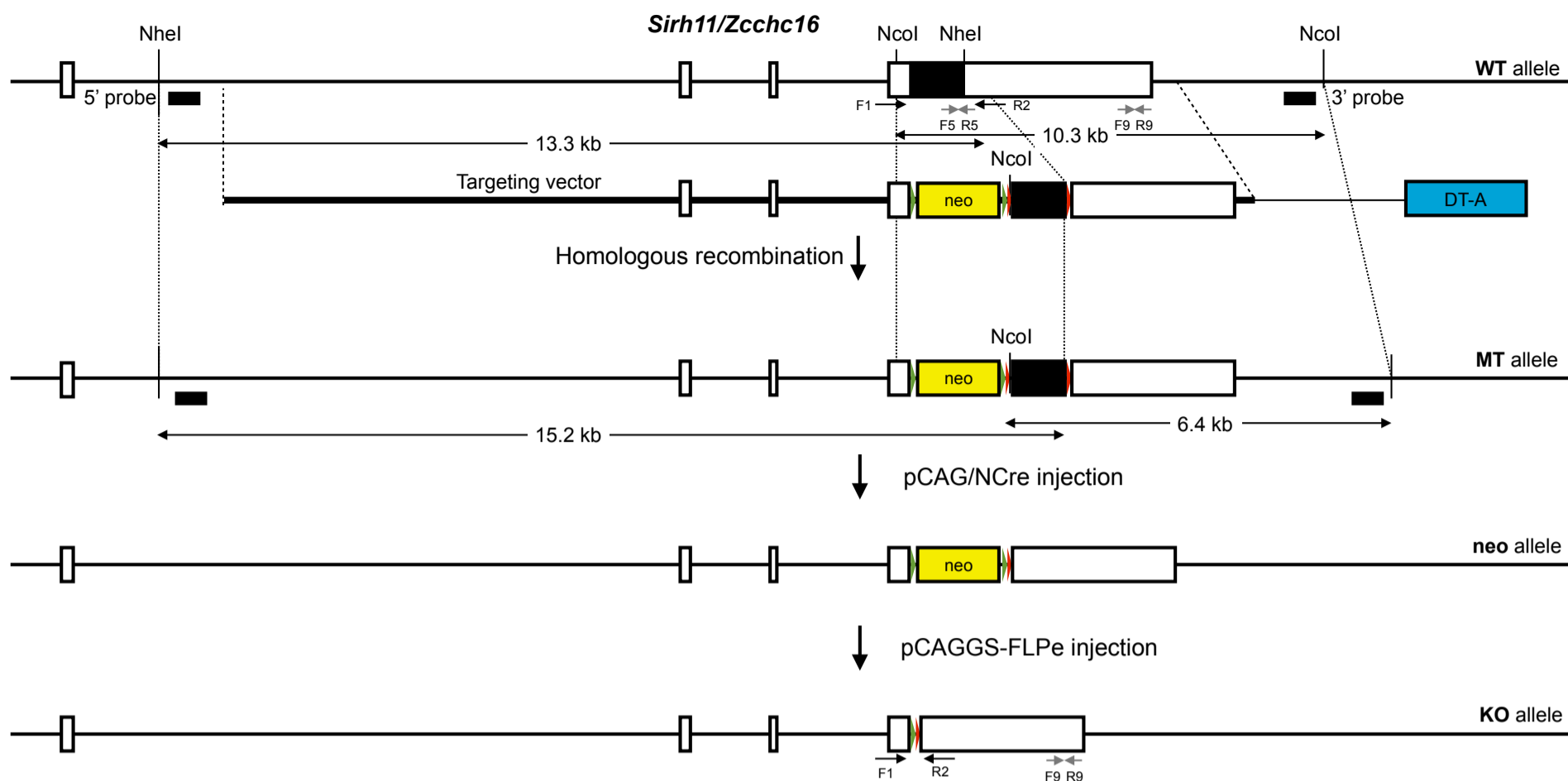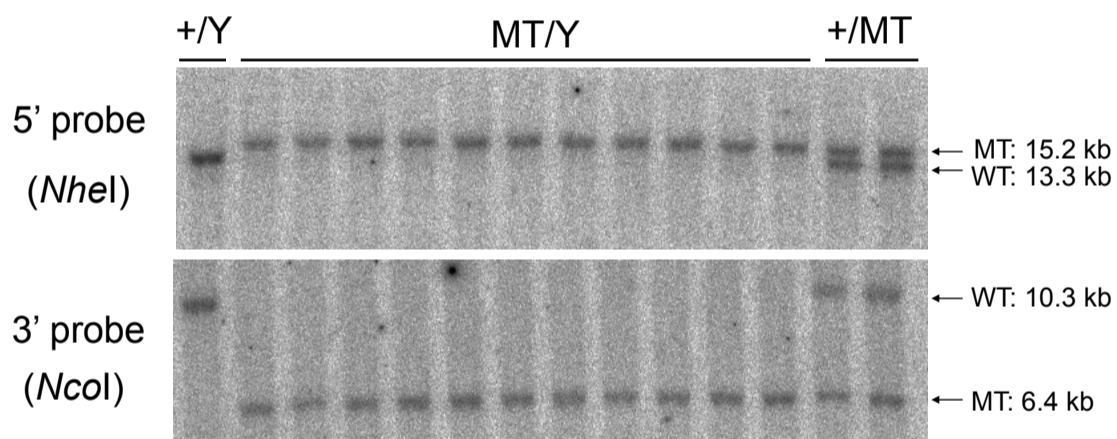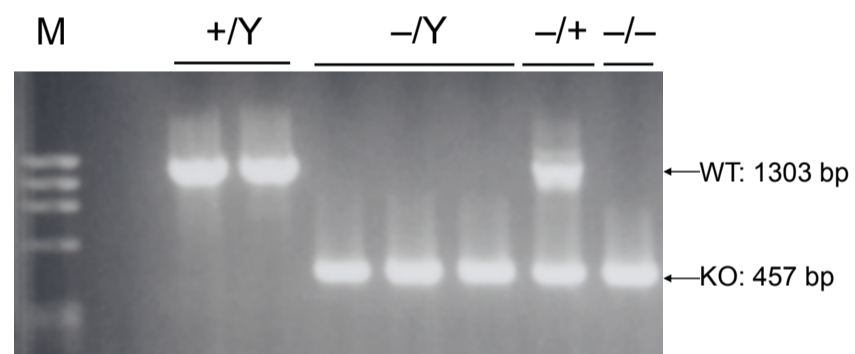

Supplement: S2 Fig — Schematic representation of the WT Sirh11 locus (WT allele), the targeting vector and targeted Sirh11/Zcchc16 allele (MT allele), the Sirh11 ORF allele (black) deleted by Cre recombinase injection (neo allele) and the neo-cassette allele (yellow) deleted by FLPe recombinase injection (KO allele). The red and green triangles represent loxP and frt, respectively. To confirm homologous recombination, Southern blot analysis was carried out using 5’ and 3’ probes (black boxes) in Sirh11/Zcchc16 MT mice. The genotyping PCR results using the F1 and R2 primers (arrows) are shown. F5, R5, F9 and R9 primers (grey arrows) were used for the qRT-PCR experiment. F5R5 primer sets were designed in the flox region and F9R9 primer sets were in the 3’ UTR. (PDF) [file pgen.1005521.s002.pdf]

## Bilirubin

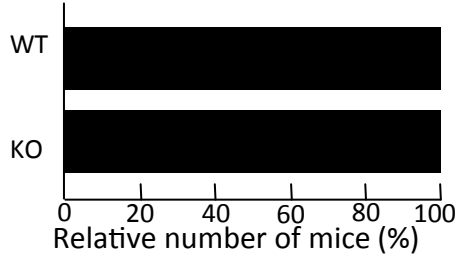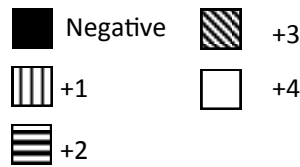

## Glucose

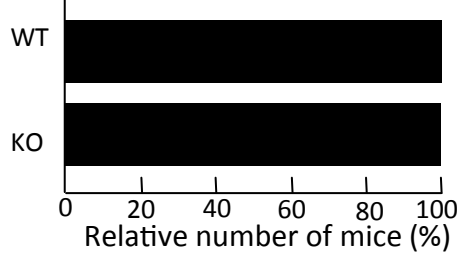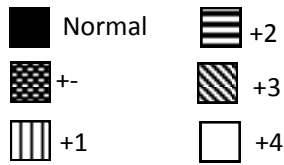

## Ketone body

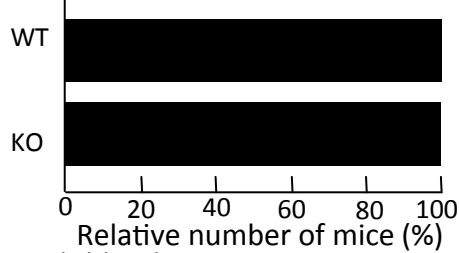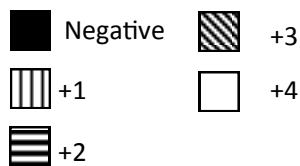

## Occult blood

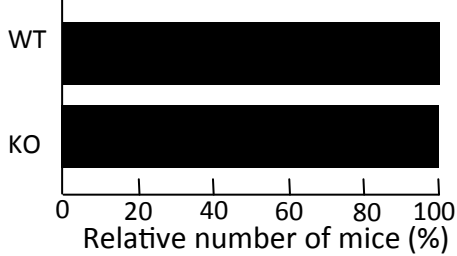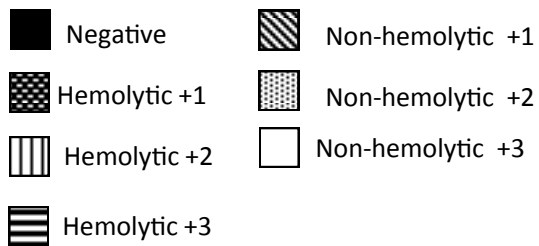

## Urine protein

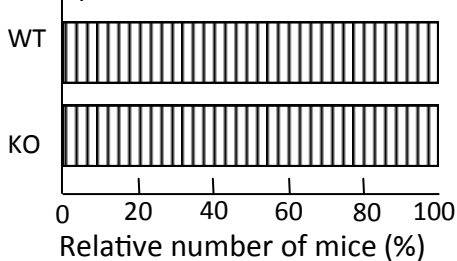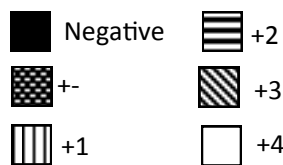

## Urobilinogen

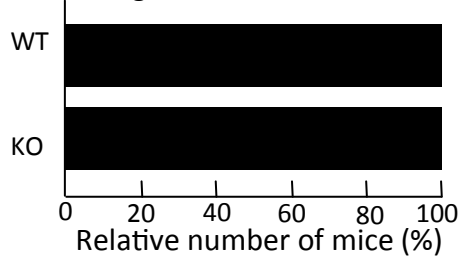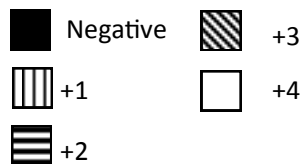

## pH

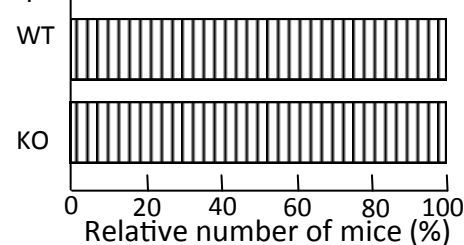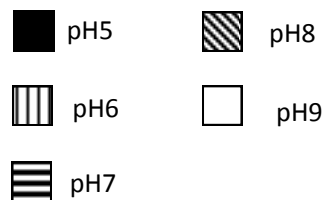

Supplement: S3 Fig — Bilirubin, glucose, ketone bodies, occult blood, urine protein and urobilinogen as well as the urine pH were examined. Urine-test papers (Aution Sticks 7EA; product code 73507) were purchased from a commercial supplier (Arkray, Tokyo, Japan). The colors of test papers that were immersed to urine were classified by comparing them with standard colors. The horizontal axis of the graph indicates the percentage of mice that exhibited each of the values shown on the right side. The male mice were tested at 10w (N = 7/genotype). There was no significant difference detected between the wild type and knockout mice. (PDF) [file pgen.1005521.s003.pdf]

WT

KO

8 w

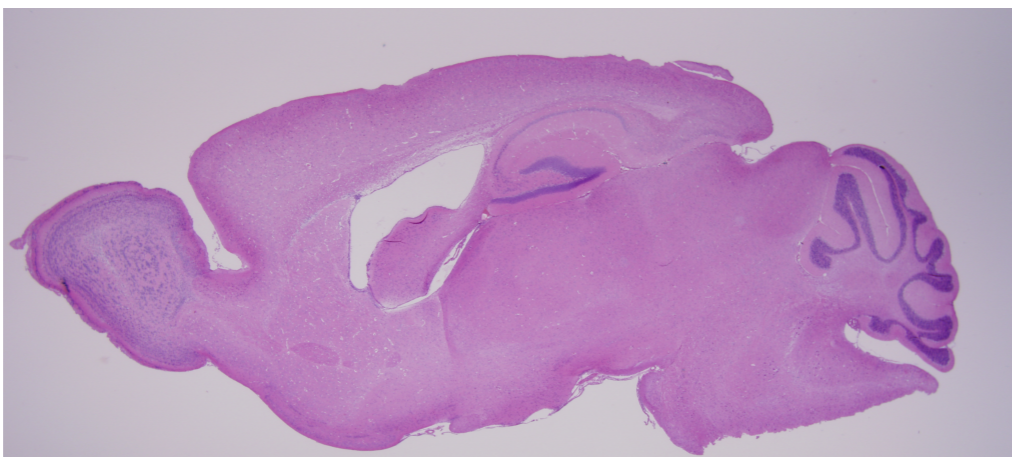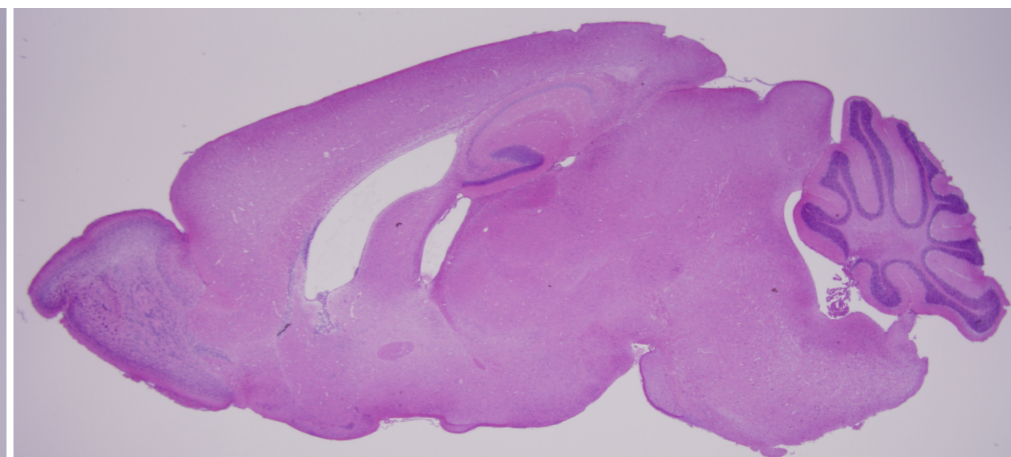

12 w

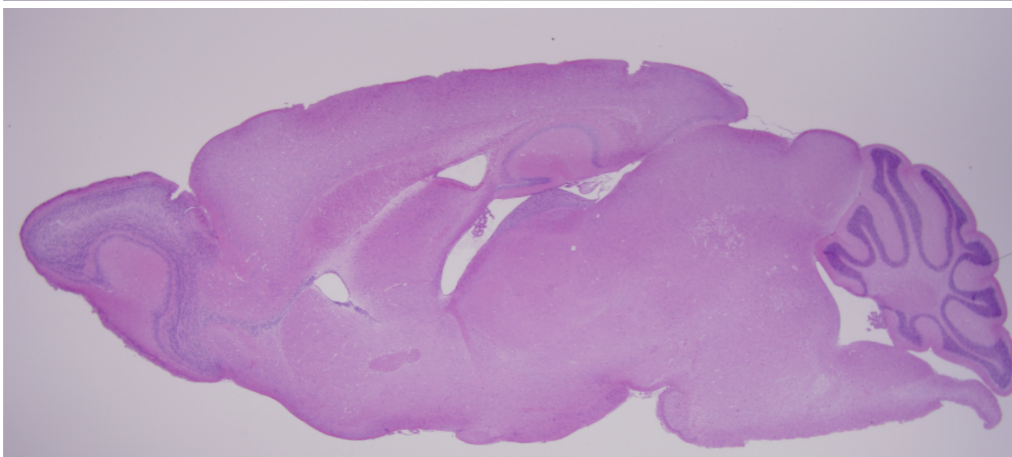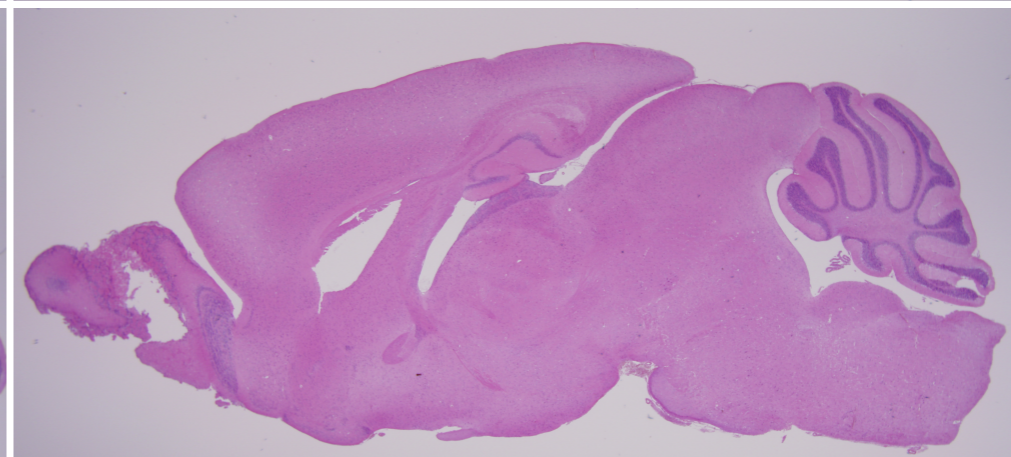

16 w

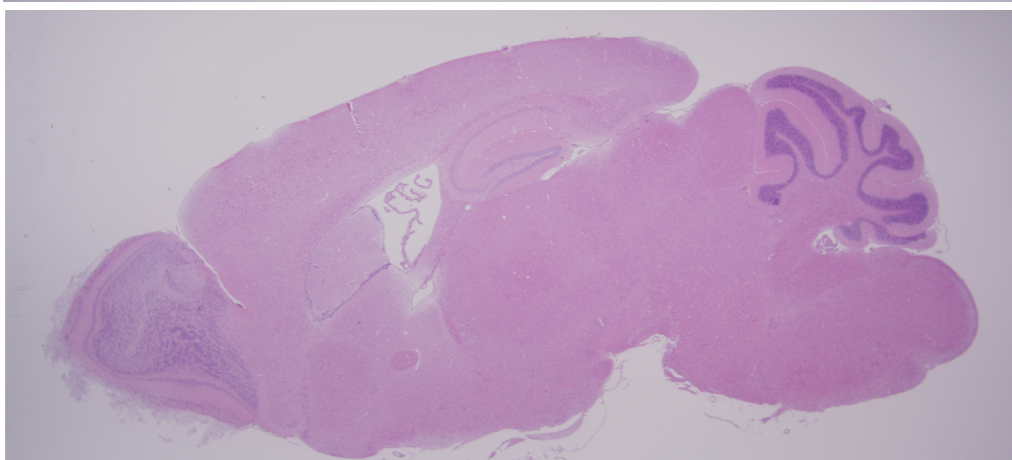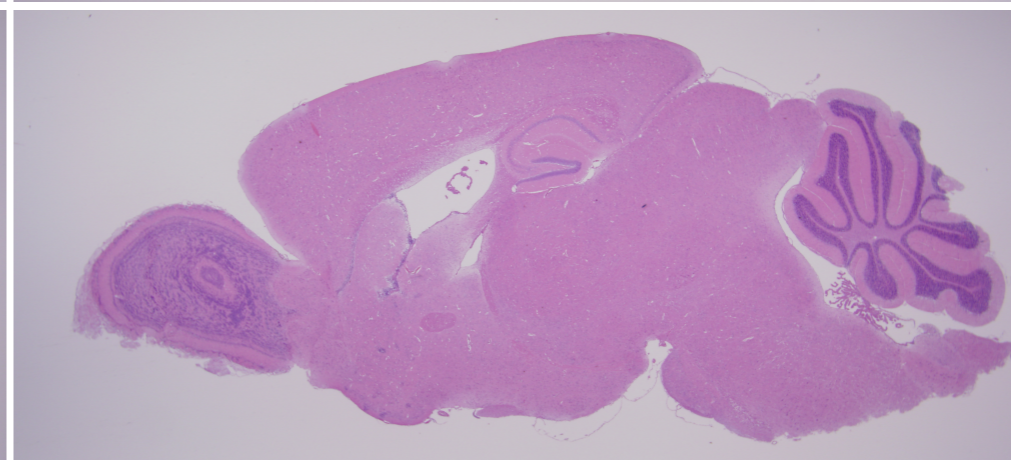

1.0 mm

Supplement: S4 Fig — The brain was isolated from male mice at 8, 12 and 16 w (N = 3 each). The brain was fixed in 4% PFA/PBS overnight and embedded in paraffin. A series of sagittal sections was stained with hematoxylin-eosin (HE). The scale bar represents 1.0 mm. No apparent differences in overall brain structure between the WT and KO were found in any of the stages. (PDF) [file pgen.1005521.s004.pdf]
